# Premature polyadenylation of *MAGI3* is associated with diminished N^6^-methyladenosine in its large internal exon

**DOI:** 10.1038/s41598-018-19916-8

**Published:** 2018-01-23

**Authors:** Thomas K. Ni, Jessica S. Elman, Dexter X. Jin, Piyush B. Gupta, Charlotte Kuperwasser

**Affiliations:** 1 Department of Developmental, Chemical and Molecular Biology, Tufts University School of Medicine, 136 Harrison Ave, Boston, MA 02111 USA; 2 Raymond & Beverly Sackler Convergence Laboratory, Tufts University School of Medicine, 136 Harrison Ave, Boston, MA 02111 USA; 3 Molecular Oncology Research Institute, Tufts Medical Center, 800 Washington St, Boston, MA 02111 USA; 4 Whitehead Institute for Biomedical Research, Cambridge, MA 02142 USA; 5 Department of Biology, Massachusetts Institute of Technology, Cambridge, MA 02139 USA

## Abstract

In cancer, tumor suppressor genes (TSGs) are frequently truncated, causing their encoded products to be non-functional or dominant-negative. We previously showed that premature polyadenylation (pPA) of *MAGI3* truncates the gene, switching its functional role from a TSG to a dominant-negative oncogene. Here we report that *MAGI3* undergoes pPA at the intron immediately downstream of its large internal exon, which is normally highly modified by N^6^-methyladenosine (m^6^A). In breast cancer cells that upregulate *MAGI3*^*pPA*^, m^6^A levels in the large internal exon of *MAGI3* are significantly reduced compared to cells that do not express *MAGI3*^*pPA*^. We further find that *MAGI3*^*pPA*^ transcripts are significantly depleted of m^6^A modifications, in contrast to highly m^6^A-modified full-length *MAGI3* mRNA. Finally, we analyze public expression data and find that other TSGs, including *LATS1* and *BRCA1*, also undergo intronic pPA following large internal exons, and that m^6^A levels in these exons are reduced in pPA-activated breast cancer cells relative to untransformed mammary cells. Our study suggests that m^6^A may play a role in regulating intronic pPA of *MAGI3* and possibly other TSGs, warranting further investigation.

## Introduction

Polyadenylation is an essential process controlling gene expression, yet how cancer cells deregulate this process to drive malignancy is only beginning to be appreciated. Polyadenylation requires *cis*-acting RNA sequence elements, most notably the AAUAAA sequence motif known as the poly(A) signal (PAS), which is recognized by *trans*-acting cleavage and polyadenylation proteins^1^. The AAUAAA motif is fairly ubiquitous and, besides its presence in terminal exons, can frequently be found in introns. Typically, intronic PAS are prevented from triggering cleavage and polyadenylation by ribonucleoprotein complexes that bind to suppressive RNA sequence elements, such as U1 snRNA-binding sites^2,3^. Despite these molecular safeguards, we previously showed that instances of intronic PAS activation do occur in cancer^4^. For example, in the MDA-MB-231 human breast cancer cell line and in primary human breast tumors, we found that oncogenic truncations of MAGI3 (MAGI3^pPA^) are caused by premature polyadenylation (pPA) triggered by intronic PAS activation^4^. We also previously characterized MAGI3^pPA^ and found that this truncation interfered with the ability of full-length MAGI3 to bind and inactivate YAP, thereby promoting malignant transformation in breast cancer cells by functioning in a dominant-negative manner. However, the molecular mechanism that activates pPA of *MAGI3* remains unknown since no *cis*-acting mutations were found in the gene^4^. In addition, it is unclear how and why pPA of *MAGI3* occurs specifically in intron 10 but not in any of the other nineteen introns of the gene, most of which also harbor cryptic PAS.

In principle, imbalances in *trans*-acting factors could give rise to *MAGI3*^*pPA*^. In practice however, because such factors participate widely in PAS recognition, changes in their activity impart widespread consequences on the polyadenylation of most multi-exon genes. The result is the production of many pPA-truncated products per gene; yet this is not observed for the 21-exon *MAGI3*^4^. Indeed, depletion of U1 snRNP, which protects pre-mRNAs from pPA, results in activation of multiple intronic PAS in the 5′ regions of most genes^3^, with a strong bias for PAS in intron 1^5^. These results cannot account for the focal pPA event occurring in intron 10 of *MAGI3*^4^, yet not in upstream introns that are more likely to be affected by *trans*-acting factors. Intrigued by the specific occurrence of pPA following exon 10 of *MAGI3* but not following other exons of the gene, we hypothesized that novel *cis*-acting elements may mark and render this gene region, and possibly others like it, susceptible to focal pPA events.

## Results

### Intronic pPA of *MAGI3* occurs following the gene’s large internal exon

**Figure 1:**
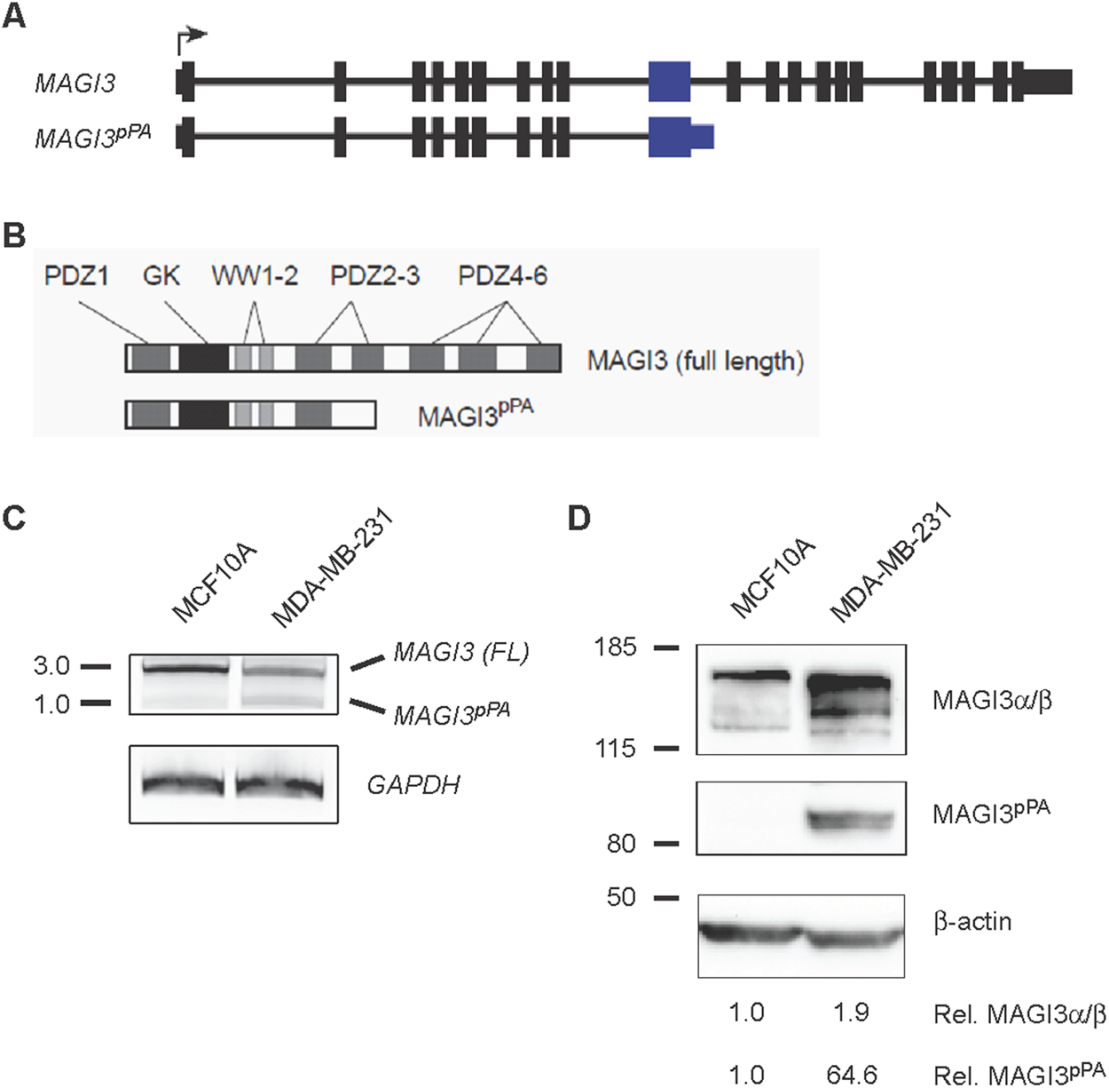
Intronic pPA of *MAGI3* occurs following the gene’s large internal exon. (**A**) Diagrams showing the exon/intron arrangement of the full length *MAGI3* gene and its truncated variant, *MAGI3*^*pPA*^. The large internal exon is colored blue. (**B**) Domains of the encoded gene products are shown for full-length MAGI3 and MAGI3^pPA^. (**C**) Full-length *MAGI3* and truncated *MAGI3*^*pPA*^ mRNA are detected in the MDA-MB-231 human breast cancer cell line but not the non-transformed MCF10A human mammary cell line by 3′ RACE. Amplification of *GAPDH* is included to show loading for 3′ RACE and approximate molecular mass markers are indicated in kb. (**D**) Full length MAGI3 and truncated MAGI3^pPA^ proteins are detected by immunoblotting. Immunoblot of β-actin is included to show loading, approximate molecular mass markers are indicated in kDa, and the relative levels of full-length and pPA-truncated MAGI3 proteins were normalized to β-actin levels.

To understand the focal nature of pPA in the *MAGI3* gene, we first examined the structure of the entire gene. *MAGI3* is a large gene comprised of 21 exons (Fig. 1A). As reported more extensively in our previous work^4^, breast cancer-associated pPA of *MAGI3* occurs in intron 10, following exon 10 (Fig. 1A). This event leads to the expression of a truncated, dominantly-acting oncogene (Fig. 1B), which can be detected by both 3′ rapid amplification of cDNA ends (RACE) and immunoblotting in MDA-MB-231 human breast cancer cells but not in MCF10A non-transformed human mammary cells (Fig. 1C and D)^4^. Upon examining the gene structure, we noticed that the occurence of pPA in intron 10 might be particularly significant since the preceding exon is by far the largest in the gene at 606 nt. The size of exon 10 is additionally noteworthy because large internal exons are a rare class of exons in the genome, likely because efficient splicing favors exon sizes less than 200 nt^6^. Thus, we hypothesized that a molecular mechanism which normally limits the usage of the intronic PAS downstream of the large internal exon of *MAGI3* may be deregulated in cancer.

### N^6^-methyladenosine (m^6^A) is normally enriched in the large internal exon of *MAGI3*, but its levels are reduced in pPA-activated MDA-MB-231 cells

To begin to test our hypothesis, we asked whether molecular marks enriched in large internal exons might correlate with the expression of *MAGI3*^*pPA*^. Interestingly, studies examining methylation of mRNA at N^6^-adenosine (N^6^-methyladenosine or m^6^A) on a transcriptome-wide scale have previously reported consistent enrichment of m^6^A in large internal exons as well as terminal exons across several human cell lines^7,8^. While the functional significance of these modifications in large internal exons has remained unclear, m^6^A density in terminal exons has been found to correlate inversely with proximal PAS usage in 3′ UTR alternative polyadenylation^9^. These data raise the possibility that m^6^A may influence the usage of proximal downstream PAS.

**Figure 2:**
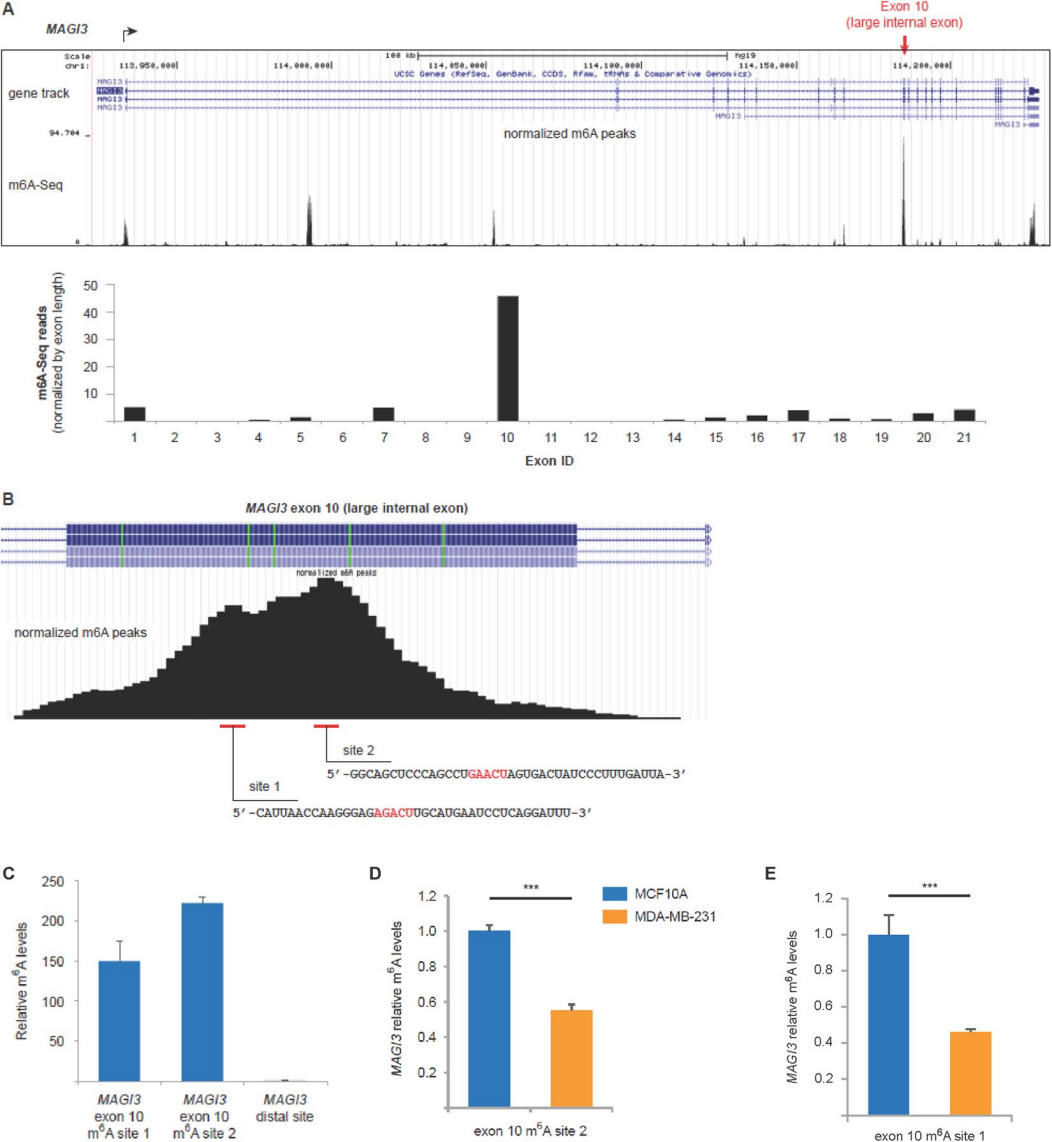
The large internal exon of *MAGI3* is highly modified by m^6^A in HEK293T, HepG2 and MCF10A cells but shows diminished m^6^A levels in pPA-activated MDA-MB-231 cells. (**A**) Distribution of m^6^A-Seq peaks across the *MAGI3* gene locus, based on analysis of previously published m^6^A-Seq data in HepG2 cells^7^. Peak number and positions in HepG2 cells were found to be highly concordant with those found in HEK293T cells by an independent m^6^A-Seq study^8^. Below, the normalized number of m^6^A-Seq reads mapping to each exon of *MAGI3* is plotted. (**B**) Distribution of m^6^A-Seq peaks across the large internal exon of *MAGI3*, exon 10. The locations and sequences of putative m^6^A sites within the large internal exon are indicated. (**C**) m^6^A levels at the indicated m^6^A consensus sites of *MAGI3*, relative to a distal *MAGI3* exonic segment (exons 1–2), as determined by m^6^A RIP-qPCR in MCF10A cells (n = 3 m^6^A RIP replicates). (**D**,**E**) Relative m^6^A levels at the indicated m^6^A consensus sites of *MAGI3* large internal exons, as determined by m^6^A RIP-qPCR in MCF10A and MDA-MB-231 cells (n = 3 m^6^A RIP replicates). Data in (**C**–**E**) are presented as mean ± SEM. ***p ≤ 0.001 (two-tailed Student’s t-tests).

Interrogating two transcriptome-wide m^6^A sequencing (m^6^A-Seq) datasets generated in the human hepatocellular carcinoma HepG2 and non-malignant human embryonic kidney HEK293T cell lines^7,8^, we found strong enrichment of m^6^A peaks in the large internal exon of *MAGI3* (Fig. 2A). Notably, the concordance between the m^6^A peaks found in HepG2 and HEK293T cells was very strong. By normalizing the number of m^6^A reads to exon length, we observed that the vast majority of m^6^A marks in the *MAGI3* mRNA are contained in the large internal exon (Fig. 2A).

Previous work has identified the m^6^A consensus sequence RRACU, where R is either G or A^7,8^. In the 606-nt large internal exon of *MAGI3*, we found only two RRACU sequences, each positioned at the center of the two observed m^6^A-Seq peaks (Fig. 2B). To validate m^6^A presence in the *MAGI3* large internal exon, we used a m^6^A-specific antibody to perform RNA immunoprecipitation (RIP) on ~100-nt chemically fragmented, poly(A)-purified RNA from MCF10A mammary epithelial cells. Relative methylation levels of fragments containing m^6^A consensus sites in the large internal exon of *MAGI3* were determined by real-time PCR (qPCR) using flanking primers. To confirm the specificity of m^6^A RIP-qPCR, we included as negative controls primers flanking exonic regions (exons 1–2) of *MAGI3* located far from m^6^A consensus sites (distal mRNA segments). Indeed, after we performed m^6^A RIP-qPCR, immunoprecipitated mRNA fragments containing the m^6^A consensus sites of the *MAGI3* large internal exon were detected at high levels, whereas distal mRNA fragments were hardly detected at all (Fig. 2C).

We next focused on validating that m^6^A modifications at the two identified sites in the large internal exon of *MAGI3* functionally promotes interaction with known m^6^A-binding proteins. Thus, we synthesized two biotinylated RNA moieties spanning each site, one m^6^A-modified within the RRACU motif and the other unmodified. Following incubation with MCF10A nuclear lysates, we immunoprecipitated the synthesized RNA by streptavidin-bound beads and performed mass spectrometry analysis (RIP-MS) on the bound samples. This analysis yielded three proteins enriched in the m^6^A-modified RIP samples of each site, including the m^6^A-binding proteins YTHDF1 and YTHDF3 (Table 1), thereby demonstrating that m^6^A modification at either site of *MAGI3* exon 10 functionally promotes interaction with experimentally validated m^6^A readers^10–12^. Following confirmation that m^6^A modification of *MAGI3* exon 10 is functionally significant, we asked whether m^6^A modification in this exon differed between MDA-MB-231 and MCF10A cells by performing additional m^6^A RIP-qPCR experiments. We found that the relative abundance of m^6^A at both sites in the large internal exon of *MAGI3* was significantly reduced in MDA-MB-231 compared to MCF10A cells (Fig. 2D and E).

**Table 1:** Proteins interacting with m^6^A-modified *MAGI3* exon 10 sites as identified by RIP-mass spectrometry.

Protein ID	m^6^A-modified RIP peptides, site 1	m^6^A-unmodified RIP peptides, site 1	m^6^A-modified RIP peptides, site 2	m^6^A-unmodified RIP peptides, site 2
TRRAP	10	0	6	0
YTHDF3	8	3	13	2
YTHDF1	8	3	6	1

### pPA-truncated *MAGI3* transcripts are largely depleted of m^6^A modifications

Having shown an overall reduction in large internal exon m^6^A modification for *MAGI3* in pPA-activated cancer cells, we next endeavored to determine whether this overall depletion of m^6^A marks in the large internal exon is specific to pPA-truncated transcripts or whether it occurs indiscriminately between full-length and truncated isoforms. We hypothesized that if m^6^A levels do not contribute to the activation of pPA, then full-length and pPA-truncated *MAGI3* transcripts will not differ significantly in methylation status. We modified the m^6^A RIP protocol used previously in order to test this null hypothesis by eliminating the chemical fragmentation step such that we could immunoprecipitate intact, poly(A)-purified RNA from MDA-MB-231 cells. In addition to the immunoprecipitated RNA, we also extracted mRNA from the unbound fraction. We subsequently performed 3′ RACE using *MAGI3*-specific forward primers and an oligo-d(T) reverse primer for each extracted fraction.

**Figure 3:**
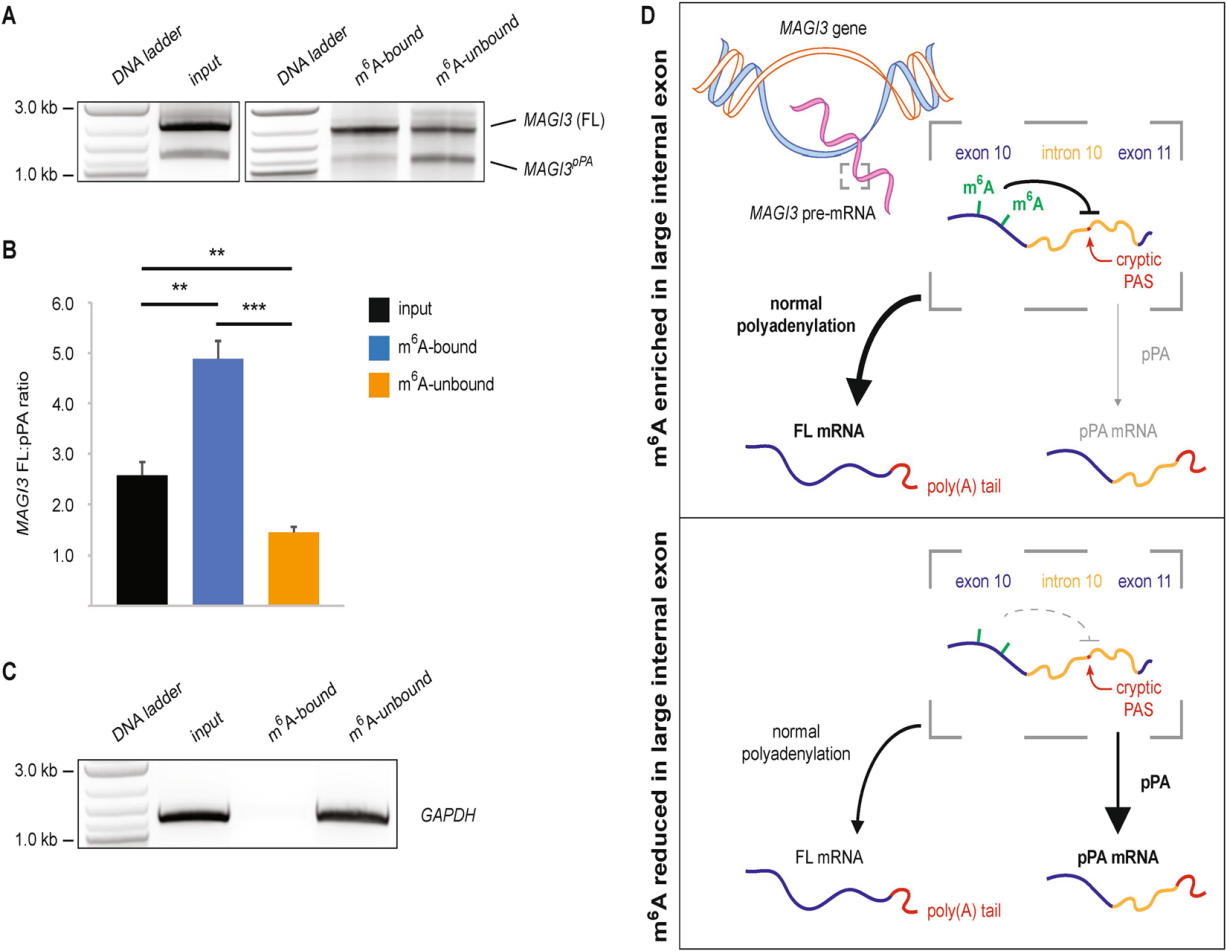
The pPA-truncated *MAGI3* isoform is predominantly unmodified by m^6^A. (**A**) Full-length and pPA-truncated *MAGI3* mRNA isoforms from MDA-MB-231 cells, fractionated into m^6^A-bound and m^6^A-unbound pools and detected by 3′ RACE (m^6^A RIP-RACE). Products from nested 3′ RACE reactions performed on MDA-MB-231 input, m^6^A-bound and m^6^A-unbound samples were separated by agarose gel electrophoresis. pPA-truncated and full-length *MAGI3* transcripts are indicated. (**B**) Ratios of full-length to pPA-truncated (FL:pPA) *MAGI3* mRNA isoforms from MDA-MB-231 input, m^6^A-bound and m^6^A-unbound fractions as detected by m^6^A RIP-RACE and quantified by densitometry using ImageJ (n = 3 technical replicates of 3′ RACE per m^6^A RIP, 2 biological replicates of the m^6^A RIP procedure). Data are presented as mean ± SEM. **p ≤ 0.01, ***p ≤ 0.001 (two-tailed Student’s t-tests). (**C**) Full-length *GAPDH* mRNA from MDA-MB-231 cells, fractionated into m^6^A-bound and m^6^A-unbound pools and detected by m^6^A RIP-RACE. Products from nested 3′ RACE reactions performed on MDA-MB-231 input, m^6^A-bound and m^6^A-unbound samples were separated by agarose gel electrophoresis. (**D**) Model for m^6^A-mediated repression of the *MAGI3* intronic PAS downstream of large internal exons. Methylation of m^6^A sites (green tick marks) in large internal exons represses cryptic intronic PAS usage in the downstream intron, favoring the generation of full-length transcripts (upper panel). Hypomethylation of m^6^A sites in large internal exons reduces the bias against downstream cryptic intronic PAS usage, leading to increased production of pPA-truncated transcripts (lower panel).

Strikingly, these experiments using m^6^A RIP-RACE revealed that pPA-truncated transcripts of *MAGI3* were significantly enriched in the unmethylated fraction and depleted from the methylated fraction (Fig. 3A and B). In contrast, full-length *MAGI3* transcripts were highly enriched in the methylated fraction, and only a minority was observed in the m^6^A-unbound fraction (Fig. 3A and B). As a control, we performed m^6^A RIP-RACE for *GAPDH*, which has no large internal exons and is not modified by m^6^A^7^. *GAPDH* transcripts were detected only in the unmethylated fraction, thus confirming the specificity of the m^6^A RIP-RACE (Fig. 3C). These data demonstrate that hypomethylation of N^6^-adenosine in the large internal exon of *MAGI3* is significantly associated with pPA-truncated, oncogenic *MAGI3* transcripts. Taken together, our data to this point suggest that depletion of m^6^A modifications from the large internal exon of *MAGI3* may somehow bias the favorability of using the downstream cryptic PAS in intron 10 (Fig. 3D). However, the generality of this proposed model remains uncertain and requires an understanding of whether other tumor suppressor genes (TSGs) also show evidence of pPA events following m^6^A-depleted large internal exons like *MAGI3*.

### Evidence of pPA events following the large internal exons of additional tumor suppressor genes

To begin addressing these questions, we investigated whether other tumor suppressor genes (TSGs) also show evidence of pPA events following large internal exons like *MAGI3*. Using public mRNA isoform expression databases to survey fifty TSGs from the Cancer Gene Census^13^, we found that twenty of them harbor at least one large internal exon (defined as >500 nt) (Supplementary Table S1). Of these, seven TSGs (*ATRX*, *BCOR*, *BRCA1*, *BRCA2*, *LATS1*, *MSH6* and *RNF43*) have previously annotated mRNA isoforms terminating in introns immediately following large internal exons (Table 2). As a caveat, we note that having identified truncations arising from pPA in these seven TSGs does not preclude the possibility that the other thirteen TSGs in the list might also undergo intronic pPA following large internal exons. These data suggest that pPA may act as a more common mechanism for truncating TSGs than previous appreciated.

**Table 2:** TSGs with large internal exons truncated by pPA in immediate downstream introns.

Gene ID	Large Internal Exon(s)	FL Exons	pPA Exons	pPA UCSD ID	pPA RefSeq ID
*ATRX*	9 (3.1 kb)	35	9	uc010nly.1	n/a
*BCOR*	4 (2.8 kb)	15	4	uc004deq.4	n/a
*BRCA1*	10 (3.4 kb)	24	10	uc002idd.5	n/a
*BRCA2*	10 (1.1 kb)/11 (4.9 kb)	27	10	uc001uua.1	n/a
*LATS1*	4 (1.5 kb)/5 (0.6 kb)	8	4	uc003qmw.4	NM_001270519.1
*MAGI3*	10 (0.6 kb)	21	10	n/a	n/a
*MSH6*	4 (2.5 kb)	10	4	uc002rwc.2	n/a
*RNF43*	9 (1.4 kb)	10	9	uc010dcw.3	n/a

**Figure 4:**
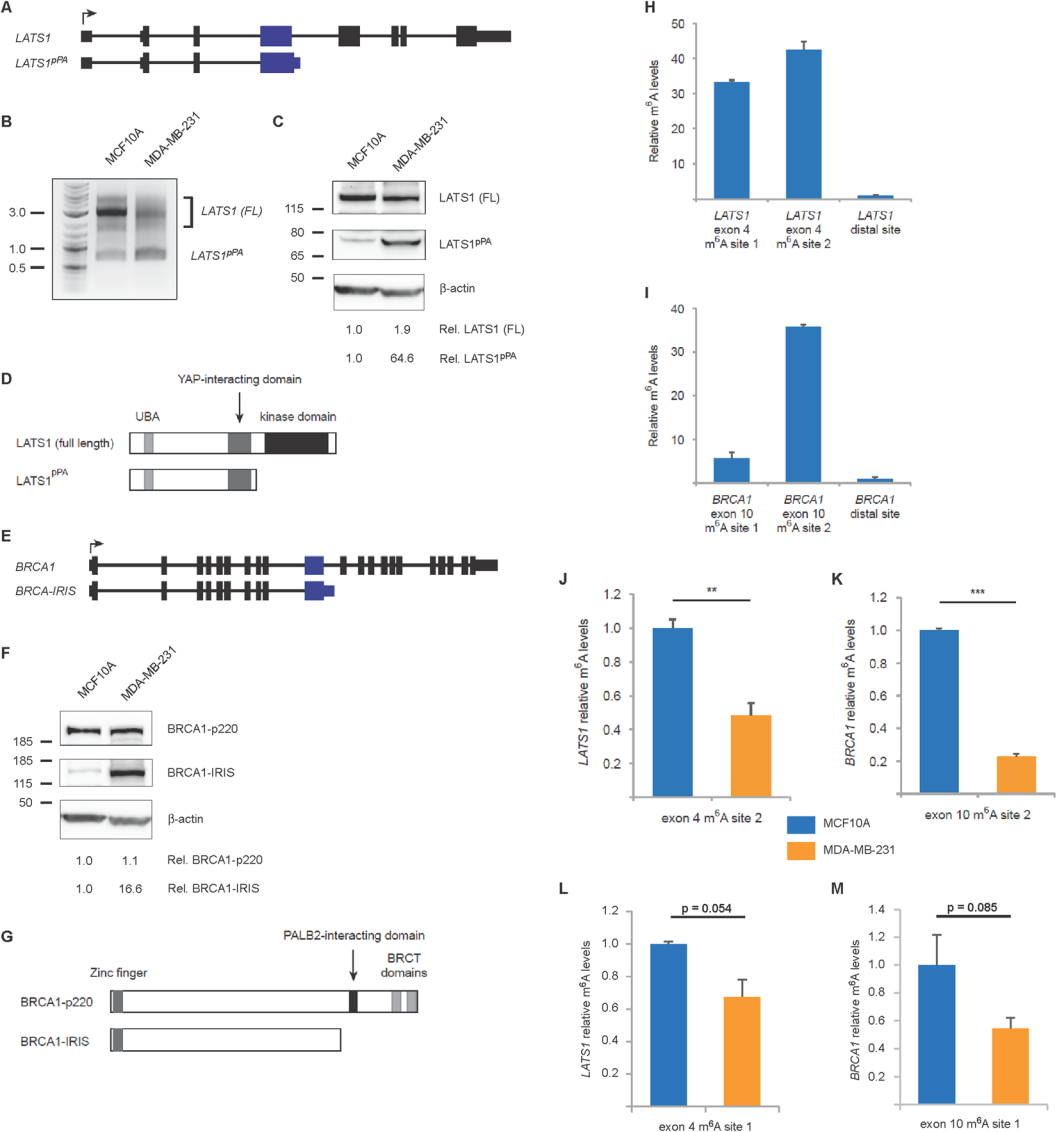
Intronic pPA events occur following the large internal exons of additional TSGs and correlate with reduced large internal exon m^6^A levels. (**A**) Diagrams showing the exon/intron arrangement of the full-length *LATS1* gene and a truncated variant. The large internal exon is colored blue. (**B**) Detection of full-length *LATS1* mRNA isoforms (lengths vary depending on 3′ UTR PAS selection) as well as a truncated *LATS1*^*pPA*^ mRNA isoform corresponding to intronic pPA downstream of exon 4 in the MDA-MB-231 and MCF10A cell lines by 3′ RACE. Approximate molecular mass markers are indicated in kb. (**C**) Immunoblot of LATS1 full-length and pPA-truncated products in the indicated cell lines. The membrane from Fig. 1D was stripped and re-probed with an anti-LATS1 antibody. Immunoblot of β-actin is included to show loading, approximate molecular mass markers are indicated in kDa, and the relative levels of full-length and pPA-truncated LATS1 proteins were normalized to β-actin levels. (**D**) Domains and functional regions of the encoded LATS1 full-length and pPA-truncated proteins. (**E**) Diagrams showing the exon/intron arrangement of the full-length *BRCA1* gene and a truncated variant. The large internal exon is colored blue. (**F**) Immunoblots of BRCA1-p220 and BRCA1-IRIS proteins in the indicated cell lines. Immunoblot of β-actin is included to show loading, approximate molecular mass markers are indicated in kDa, and the relative levels of full-length and pPA-truncated BRCA1 proteins were normalized to β-actin levels. (**G**) Domains and functional regions of the encoded gene products, BRCA1-p220 and BRCA1-IRIS. (**H**) m^6^A levels at the m^6^A consensus sites of *LATS1*, relative to a distal *LATS1* exonic segment (exons 2–3), as determined by m^6^A RIP-qPCR in MCF10A cells (n = 3 m^6^A RIP replicates). (**I**) m^6^A levels at the m^6^A consensus sites of *BRCA1*, relative to a distal *BRCA1* exonic segment (exons 2–3), as determined by m^6^A RIP-qPCR in MCF10A cells (n = 3 m^6^A RIP replicates). (**J**–**M**) Relative m^6^A levels at the indicated m^6^A consensus sites of *LATS1* (**J**,**L**) and *BRCA1* (**K**,**M**) large internal exons, as determined by m^6^A RIP-qPCR in the indicated cell lines (n = 3 m^6^A RIP replicates). Data in (**H**–**J**) are presented as mean ± SEM. **p ≤ 0.01, ***p ≤ 0.001 (two-tailed Student’s t-tests).

Among the seven TSGs showing evidence of pPA, the truncated *LATS1* isoform is particularly similar to *MAGI3*^*pPA*^ since previous studies have suggested that truncation products of *LATS1* act to functionally oppose its tumor suppressive function. In the *LATS1* gene, pPA occurs at a cryptic PAS in intron 4, following the 1.5-kb exon 4 (Fig. 4A)^14^. This pPA-truncated transcript of *LATS1* was identified in a candidate full-ORF cDNA library generated from a variety of cellular sources^14^, and has not been extensively studied since its initial annotation. Thus we performed 3′ RACE to validate its expression specifically in the “pPA-activated” MDA-MB-231 breast cancer cell line and non-transformed MCF10A cell line. Indeed by 3′ RACE, we observed an upregulation of the truncated LATS1 mRNA in MDA-MB-231 compared to MCF10A cells, apparently at the expense of full-length LATS1 levels (Fig. 4B). By immunoblotting with an antibody raised against the N-terminal region of LATS1, we also found upregulation of LATS1^pPA^ in MDA-MB-231 compared to MCF10A cells (Fig. 4C). Interestingly, the truncated LATS1 isoform (hereafter LATS1^pPA^) lacks the kinase domain necessary for suppressing oncogenic YAP activity but retains the YAP-interacting domain (Fig. 4D). Overexpression of experimentally truncated LATS1 products of similar length to LATS1^pPA^ has been reported to dominantly interfere with LATS1-mediated regulation of the centrosome during mitosis, thus promoting mitotic delay and tetraploidy^15,16^, and additionally bind to full-length LATS1 proteins in an inhibitory manner^17,18^. Taken together, these data suggest that MDA-MB-231 breast cancer cells may have positively selected for the pPA-truncated product of LATS1 as a potentially oncogenic protein variant.

We also looked at *BRCA1* as another example of the seven TSGs showing evidence of pPA. Following the 3.4-kb exon 10, the *BRCA1-IRIS* isoform is prematurely polyadenylated downstream of a close variant of the canonical PAS (AGUAAA) in intron 10 (Fig. 4E)^19^. The expression of this truncated mRNA isoform has previously been extensively characterized by 3′ RACE, sequencing, RT-PCR and Northern blot analysis^19^. We immunoblotted MDA-MB-231 and MCF10A cell lysates with an antibody recognizing the N-terminal region of BRCA1 and observed that BRCA1-IRIS was present at higher levels in MDA-MB-231 versus MCF10A cells (Fig. 4F). BRCA1-IRIS lacks key functional regions, such as the BRCT domains and protein-interacting regions (Fig. 4G), and its expression has been previously reported to promote growth factor-independent cell proliferation, anchorage-independent colony formation, and subcutaneous tumor xenograft growth^19–22^.

### Reduced m^6^A modification of *LATS1* and *BRCA1* large internal exons in MDA-MB-231 cells

To investigate whether the large internal exons of TSGs are also typically enriched in m^6^A modifications, we again examined transcriptome-wide m^6^A sequencing (m^6^A-Seq) datasets^7,8^. Consistent with the pattern of m^6^A modification for the large internal exon of *MAGI3*, we found enrichment of m^6^A peaks in the large internal exons of *LATS1* and *BRCA1*, as well as other TSGs (Supplementary Fig. S1). It is worth noting that the complexity of m^6^A modification patterns increased with greater internal exon lengths, and the largest internal exons frequently exhibited multiple, strong m^6^A peaks with additional, weaker m^6^A peaks throughout. After identifying putative large internal exon m^6^A sites by finding the consensus sequence RRACU in the strongest m^6^A peak regions of *LATS1* and *BRCA1*, we performed m^6^A RIP-qPCR in MCF10A cells to validate the presence of m^6^A modifications. We validated m^6^A modifications in the two strongest peaks of *LATS1* exon 4, with the downstream site exhibiting the highest modification level (Fig. 4H). Meanwhile, for *BRCA1* exon 10, we validated high levels of m^6^A modification in the most downstream site, but the upstream site showed much weaker enrichment by m^6^A RIP (Fig. 4I).

We subsequently asked whether MDA-MB-231 cells differ in the levels of m^6^A modification in TSG large internal exons compared to MCF10A cells. We found that the relative abundance of m^6^A at the strongest, most downstream sites in the large internal exons of *LATS1* and *BRCA1* was significantly reduced in MDA-MB-231 cells (Fig. 4J and K), accompanied by less dramatic reductions at weaker upstream m^6^A sites (Fig. 4L and M). These data suggest that like *MAGI3*, reduced m^6^A levels in the large internal exons of *LATS1* and *BRCA1* also correlate with intronic pPA following large internal exons.

### Overall m^6^A levels and expression levels of m^6^A-modifying enzymes are comparable between MDA-MB-231 and MCF10A cells

Because we observed pPA-associated m^6^A hypomethylation in the large internal exon of *MAGI3*, as well as a general reductions in large internal exon m^6^A levels for *BRCA1* and *LATS1*, we asked whether this phenomenon might be caused by an overall reduction in m^6^A levels transcriptome-wide in pPA-activated MDA-MB-231 cells compared to pPA-protected MCF10A cells. Thus we performed dot blot assays on purified poly(A) RNA from each cell line. These experiments showed that overall levels of m^6^A modification in the two cell lines are comparable (Supplementary Figure S2A and B). We further examined whether the expression levels of genes encoding known m^6^A methyltransferase components (writers) or demethylase proteins (erasers) differ dramatically between MDA-MB-231 and MCF10A cells. We therefore assessed the expression levels of m^6^A writers *METTL3*, *METTL14* and *WTAP*^23–26^, as well as the expression levels of the m^6^A erasers *FTO* and *ALKBH5*^27,28^, in MDA-MB-231 and MCF10A cells by qPCR (Supplementary Figures S2C–G). Overall, we found that the expression levels of m^6^A-modifying enzymes were comparable between the two cell lines (Supplementary Figures S2C–G), with only slight differences observed. Moreover, when m^6^A writers or erasers were considered together as functional groups, we did not observe collective trends in one cell line versus the other. For instance, while *METTL3* levels were slightly higher in MDA-MB-231 cells, the other two m^6^A methyltransferase components, *METTL14* and *WTAP*, were expressed at slightly lower levels compared to MCF10A cells (Supplementary Figures S2C–E). Similarly, of the two m^6^A demethylases, *FTO* was expressed slightly more highly in MDA-MB-231 cells while *ALKBH5* was expressed slightly more highly in MCF10A cells (Supplementary Figures S2F and G). Taken together with the results from dot blot assays, the overall levels of m^6^A modification and the expression levels of m^6^A-modifying enyzmes do not necessarily distinguish the pPA-activated cell line, MDA-MB-231 from the non-transformed MCF10A cell line.

## Discussion

The molecular mechanism underlying cancer-associated, intronic premature polyadenylation of *MAGI3* has remained unknown because no *cis*-acting genetic mutations were found in the gene, making it unclear how pPA of *MAGI3* can specifically be activated in one intron but not in other introns that also harbor cryptic PAS^4^. In this study, we have identified N^6^-methyladenosine as a *cis*-acting epitranscriptomic mark associated with *MAGI3* mRNA shortening. We have found that *MAGI3* is affected by pPA at the intron immediately downstream of its single, large internal exon. The large internal exon of *MAGI3* is by far the most highly m^6^A-modified exon in the gene, and we have shown by RIP-MS that the lack of m6A modification at the two m^6^A consensus sites in the exon diminishes the frequency of physical interactions between the mRNA and m^6^A-reading proteins. Furthermore, we have discovered that *MAGI3*^*pPA*^ transcripts are largely depleted of m^6^A modifications while full-length *MAGI3* mRNA remains highly m^6^A-modified.

Since its discovery, the functional impact of high m^6^A levels in the large internal exons of genes has remained unclear^7^. By identifying m^6^A as a *cis*-acting epitranscriptomic mark associated with *MAGI3* mRNA shortening, we have drawn an unexpected connection between large internal exon m^6^A modifications in *MAGI3* and the expression of cancer-associated, pPA-truncated *MAGI3* transcripts. How cancer cells modulate m^6^A levels in the *MAGI3* large internal exon to trigger pPA, and how this modulation of levels impacts pPA of *MAGI3* from a mechanistic standpoint, are new questions that require further investigation. Regarding the former, several m^6^A-modifying enzymes have been recently identified, and alterations in some of these components, especially the m^6^A demethylase *FTO*, have been observed to correlate with human cancer risk^29,30^. For the latter, a bias against pPA of *MAGI3* rendered by m^6^A modification could be achieved via changes to the secondary structure of large internal exonic regions of the mRNA thus preventing downstream PAS recognition, or by binding of a m^6^A-binding protein that acts in concert with other protein factors to prevent intronic PAS usage, or a combination of both mechanisms. Indeed, similar mechanistic concepts regarding the structural aspects of genes and m^6^A-mediated post-transcriptional gene regulation have recently been put forth for consideration as a new paradigm for the coordination of gene expression^31,32^.

We have additionally analyzed publicly available mRNA expression data to report that intronic pPA-generated isoforms of other TSGs such as *LATS1* and *BRCA1* have been previously identified^14,19^. These findings suggest that pPA may act as a more pervasive oncogenic mechanism for truncating TSGs with large internal exons than previously appreciated. Interestingly, we have also found that m^6^A levels in the large internal exons of *LATS1* and *BRCA1* are significantly lower in pPA-activated breast cancer cells relative to untransformed mammary cells. Taken together with the experiments showing that reduced m^6^A modification is associated with pPA-shortening of *MAGI3*, these data are conceptually consistent with those of a previous study showing that m^6^A density is inversely correlated with proximal PAS usage in terminal exons^9^. Thus, it is intriguing to speculate that m^6^A modification of large internal exons may play a role in regulating intronic pPA of TSGs beyond *MAGI3*, and additional studies of broader scope investigating the relationship between m^6^A levels in large internal exons and intronic pPA-mediated mRNA truncation for other TSGs are warranted.

## Materials and Methods

### Cell Lines and Tissue Culture

The cell lines used in this study were purchased from ATCC and grown as described previously^4^.

### Immunoblotting and Dot Blot Assays

Cell lysis, SDS-PAGE and immunoblotting were performed as described previously^4^. For dot blot assays, poly(A) RNA was purified from total RNA using DynaBeads mRNA Purification Kit (ThermoFisher). Poly(A) RNA was serially diluted to 180 ng/µl, 45 ng/µl, 11.25 ng/µl. Each dilution was dotted (2.5 µl) on a BrightStar-Plus positively charged nylon membrane (Invitrogen) in duplicate. The poly(A) RNA was crosslinked to the membrane in a Stratalinker 2400 Crosslinker twice (1,200 µl joules) and the membrane was washed for 5 minutes in wash buffer (Phosphate Buffered Saline, 0.02% Tween-20) before blocking for 1 hr (Phosphate Buffered Saline, 5% Milk, 0.02% Tween-20). The membrane was incubated overnight at 4 °C in polyclonal rabbit anti-m^6^A antibody (2 µg/ml) diluted in blocking buffer. Treatment with secondary antibody was performed according to standard immunoblotting procedures and m^6^A detection was visualized using enhanced chemiluminescence. Levels of m^6^A were quantified by measuring density of dots using Fiji ImageJ. Antibodies used are: β-actin (Abcam ab6276); BRCA1 (ThermoFisher MA1-23160); BRCA1(Santa Cruz Biotechnology sc-642); LATS1 Goat Santa Cruz Biotechnology sc-9388; m6A for RIP (New England Biolabs E1610); m^6^A (Synaptic Systems 202–003); MAGI3 (Novus Biologicals NBP2-17210).

### RNA Preparation, m^6^A RIP-qPCR and m^6^A RIP-RACE

Total RNA was extracted from MCF10A and MDA-MB-231 cell pellets using the RNeasy Maxi Kit (Qiagen). Poly(A) RNA was purified from total RNA using the Oligotex Midi Kit (Qiagen). For m^6^A RIP-qPCR, RNA samples were chemically fragmented into ~100-nt length fragments by a 5 min incubation at 95 °C in NEBNext RNA fragmentation buffer from New England Biolabs (40 mM Tris-OAc, 100 mM KOAc, 30 mM Mg(OAc)_2_, pH 8.3). The fragmentation reaction was stopped with 50 mM EDTA, and one round of ethanol precipitation was performed to purify the fragmented poly(A) RNA. 3 µg fragmented poly(A) RNA was incubated for 1 hr at 4 °C with 1 µl EpiMark anti-m^6^A antibody (New England Biolabs) pre-bound to pre-washed Protein G magnetic beads in reaction buffer (150 mM NaCl, 10 mM Tris-HCl, pH 7.5, 0.1% NP-40). m^6^A-bound complexes were then washed twice in reaction buffer, followed by two washes in low salt reaction buffer (50 mM NaCl, 10 mM Tris-HCl, pH 7.5, 0.1% NP-40) and two washes in high salt reaction buffer (500 mM NaCl, 10 mM Tris-HCl, pH 7.5, 0.1% NP-40). Immunoprecipitated RNA was eluted in 30 µl Buffer RLT (Qiagen), then cleaned and concentrated using Dynabeads MyOne Silane (ThermoFisher) followed by ethanol washes. Bound RNA was eluted in 20 µl nuclease-free water and used for first-strand cDNA synthesis as described previously^4^. cDNA was also synthesized from total RNA, representing the input for m^6^A RIP. Samples were prepared for qPCR using isoform-specific or exon-specific primers. qPCR was performed in triplicate for each sample-target combination as described previously^4^. For determining gene expression, mRNA abundance was normalized to *GAPDH*. For m^6^A RIP samples, m^6^A levels of each target were normalized to overall expression levels of the target as determined by the same primer pair from total RNA. Targets spanning exons of the same gene but located far from the m^6^A sites within the large internal exons (distal mRNA segments) were also assayed. Primer sequences used for qPCR are: Forward Primer (F: 5′ to 3′), Reverse Primer (R: 5′ to 3′): BRCA1 exon 10 site 1F: TGAGTGGTTTTCCAGAAGTGA R: TCCCCATCATGTGAGTCATC; BRCA1 exon 10 site 2F: TCTCAGTTCAGAGGCAACGAR: TGGGTTTTGTAAAAGTCCATGTT; BRCA1-distal (exons 2–3) F: CGCGTTGAAGAAGTACAAAATG R:CAGGTTCCTTGATCAACTCCA; LATS1 exon 4 site 1F: GACCTGGAATGCAGAATGGT R: GCAGGGACAACATTTTGGTG; LATS1 exon 4 site 2F: GCCTGTGAAAAGTATGCGTGT R: GGCTGTGGTATCCAAGAAGG; LATS1-distal (exons 2–3) F: ACTTGCAAGCTGCTGGATTT R: TGTTGTTAGTTTTCTGAAGAGCTTG; MAGI3 exon 10 site 1F: TGGACAGTCATTAACCAAGGGA R: GCTCCAGAACCATTGCTCCT; MAGI3 exon 10 site2 F: CATCGTCAGGCAGCTCCC R: TGCAAACCCAAACCCTTTAGG; MAGI3-distal (exons 1–2) F: CGTCTCAAGACTGTGAAACCA R: GACTTAGGTAATGCCGCAATC; GAPDH F: CCATGGGGAAGGTGAAGGTC R: TAAAAGCAGCCCTGGTGACC; ALKBH5 F: TTCAAGCCTATTCGGGTGTC R: GGCCGTATGCAGTGAGTGAT; FTO F: AATCTGGTGGACAGGTCAGC R: TGCCTTCGAGATGAGAGTCA; METTL3 F: CCCACTGATGCTGTGTCCAT R: CTGCAGGAGGCTTTCTACCC; METTL14 F: TCCAAAGGCTGTCTTTCAGAGA R: GAAGTCCCCGTCTGTGCTAC; WTAP F: ACAAGCTTTGGAGGGCAAGT R: GATGTTTTCCCTGCGTGCAG.

For m^6^A RIP-RACE, RNA samples were not subjected to the fragmentation step and used directly for m^6^A-RIP. 3 µg unfragmented poly(A) RNA was incubated for 1 hr at 4 °C with 1 µl EpiMark anti-m^6^A antibody (New England Biolabs) pre-bound to pre-washed Protein G magnetic beads in reaction buffer (150 mM NaCl, 10 mM Tris-HCl, pH 7.5, 0.1% NP-40). Following this binding step, m^6^A-bound RNA (beads) and m^6^A-unbound RNA (supernatant) were reserved. The m^6^A-bound fraction was washed twice in reaction buffer, twice in low salt reaction buffer and twice more in high salt reaction buffer. Immunoprecipitated RNA was eluted in 30 µl Buffer RLT (Qiagen). The eluted m^6^A-bound RNA and the reserved m^6^A-unbound RNA were cleaned and concentrated using Dynabeads MyOne Silane (ThermoFisher) followed by ethanol washes. The bound and unbound RNA fractions were then eluted in 20 µl nuclease-free water, and 3′ RACE was performed as described previously^4^. *MAGI3* and *GAPDH* gene-specific forward primer sequences used for 3′ RACE are: GAPDH-primary CCATGGGGAAGGTGAAGGTC;GAPDH-nested GATTTGGTCGTATTGGGCGC; MAGI3-primary CTGTGTCCTCGGTCACACTC; MAGI3-nestedGTTGCTGCTACCCCTGTCAT.

### RIP-MS Analysis

Nuclear MCF10A lysates were obtained using the NE-PER kit (ThermoFisher) supplemented with protease inhibitors (Roche) and phosphatase inhibitors (Sigma), then precleared by incubating with streptavidin-conjugated magnetic beads (New England Biolabs) for 1 hr at 4 °C. 5′-biotin-labeled RNA oligonucleotides (42-nt in length with the RRACU m^6^A consensus motif in the center) were synthesized (Dharmacon). Two RNA oligonucleotide versions were synthesized for each *MAGI3* exon 10 m^6^A site, differing only in their m^6^A modification status. Precleared MCF10A nuclear lysates were incubated with 2 µg of the RNA oligonucleotides supplemented with 0.4 units/µl RNasin (Promega) for 1 hr at 4 °C. The RNA-nuclear lysate mixture was subsequently added to streptavidin-conjugated magnetic beads pre-blocked with 1% BSA and 50 µg/ml yeast tRNA (ThermoFisher) for 1 hr at 4 °C. Immunoprecipitated complexes were washed in Tris-HCl buffer (20 mM Tris-HCl, pH 7.5), and bound proteins were eluted by boiling in SDS loading buffer for 5 min. Protein samples were separated by SDS-PAGE according to standard procedures, fixed in the gel, stained with a 0.3% Coomassie Blue R250 solution, then destained overnight. Gel slices were digested with trypsin and analyzed by liquid chromatography-tandem mass spectrometry (Taplin Mass Spectrometry Facility, Harvard Medical School). The accepted list of interacting proteins was obtained by filtering out common cytoplasmic protein contaminants and setting stringency thresholds of six or greater peptides identified in m^6^A-modified RIP samples and three or fewer peptides identified in m^6^A-unmodified RIP samples. The modified RNA oligonucleotide sequences used for RIP-mass spectrometry are: MAGI3 site 1 m^6^A-modified Bi-gacagucauuaaccaagggagag(m6A)cuugcaugaauccucagg; MAGI3 site 1 m^6^A-unmodified Bi-gacagucauuaaccaagggagagacuugcaugaauccucagg; MAGI3 site 2 m^6^A-modified Bi-ucgucaggcagcucccagccuga(m6A)cuagugacuaucccuuug; MAGI3 site 2 m^6^A-unmodified Bi-ucgucaggcagcucccagccugaacuagugacuaucccuuug.

### Bioinformatic Analysis of m^6^A-Seq Data and Identification of Putative m^6^A Sites

Sequence data were downloaded from the Gene Expression Omnibus (GEO). The identifier for the GEO dataset is GSE37005^7^. Alignment data was obtained by following a previously published protocol for m^6^A-Seq analysis^33^, converted to bigWig format normalized per total filtered reads and loaded to the UCSC genome browser for downstream analyses. To identify putative m^6^A sites, the locations of RRACU motifs, where R is either G or A, were cross referenced with peak locations along each exon. For the 606-nt MAGI3 exon 10, only two sequences matching the RRACU motif were found, and their locations corresponded to the approximate center of the m^6^A peaks from m^6^A-Seq. For LATS 1 exon 4 and BRCA1 exon 10, the pattern of m^6^A peak signals was considerably more complex. This was due to the exon lengths and increased frequency of RRACU sequences. LATS1 exon 4 had nine RRACU sequences across 1.5-kb, and BRCA1 exon 10 had 24 RRACU sequences across 3.4-kb. m^6^A-Seq data showed that each exon had two highly modified sites (strong peaks). Besides the two strongest peaks, LATS1 exon 4 had three moderate-to-high signal peaks and two weak signal peaks, while BRCA1 exon 10 had four moderate signal peaks and nine weak signal peaks. The two strongest peaks within each exon were chosen for validation as weaker peaks were likely to represent low stoichiometry m^6^A modifications that would be difficult to distinguish from background noise in m^6^A RIP-qPCR.

### Statistical Analysis

Data were analyzed and compared between groups using two-tailed Student’s t-tests. A p < 0.05 was considered statistically significant.

## Electronic supplementary material

Supplementary Information
