# Supplementary material for: Premature polyadenylation of MAGI3 is associated with diminished N6-methyladenosine in its large internal exon
Source: Sci Rep. 2018 Jan 23;8:1415. doi: 10.1038/s41598-018-19916-8 (PMC5780518; doi:10.1038/s41598-018-19916-8)
Supplement: Supplementary file 1 — Supplementary Information [file 41598_2018_19916_MOESM1_ESM.pdf]

Premature polyadenylation of *MAGI3* is associated with diminished N^6-methyladenosine in its large internal exon

Thomas K. Ni[1,2,3], Jessica S. Elman[1,2,3], Dexter X. Jin[4,5], Piyush B. Gupta[4,5] and Charlotte Kuperwasser[1,2,3,*]

Affiliations:
[1] Department of Developmental, Chemical and Molecular Biology, Tufts University School of Medicine, 136 Harrison Ave, Boston, MA 02111, USA
[2] Raymond & Beverly Sackler Convergence Laboratory, Tufts University School of Medicine, 136 Harrison Ave, Boston, MA 02111, USA
[3] Molecular Oncology Research Institute, Tufts Medical Center, 800 Washington St, Boston, MA 02111, USA
[4] Whitehead Institute for Biomedical Research, Cambridge, MA 02142
[5] Department of Biology, Massachusetts Institute of Technology, Cambridge, MA 02139
[*] Correspondence to:
charlotte.kuperwasser@tufts.edu

Supplementary Figure S1. The large internal exons of pPA-truncated TSGs are enriched in m^6A modifications.

Distribution of m^6A-Seq peaks across the gene loci of multiple TSGs (*BRCA2*, *MSH6*, *BRCA1*, *ATRX* and *LATS1*) found to be truncated by pPA, based on analysis of previously published m^6A-Seq data in HepG2 cells [7]. Peak number and positions in HepG2 cells were found to be highly concordant with those found in HEK293T cells by an independent m^6A-Seq study [8]. pPA-truncated isoforms are outlined in red, and large internal exons upstream of pPA events are indicated by the arrows.

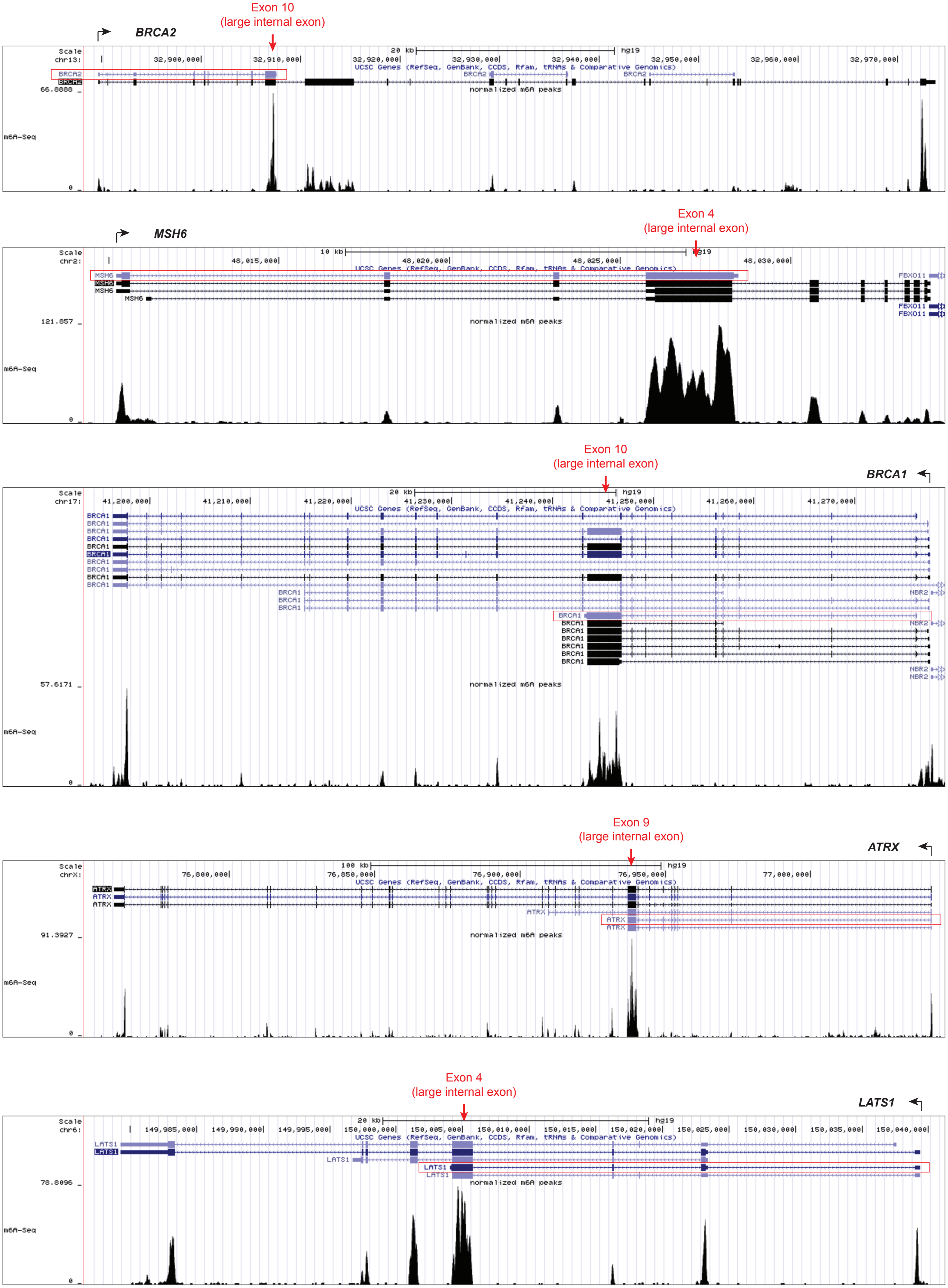
Exon 10
(large internal exon)
BRCA2
Scale
chr13:
20 kb
hg19
32,900,000
32,910,000
32,920,000
32,930,000
32,940,000
32,950,000
32,960,000
32,970,000
UCSC Genes (RefSeq, GenBank, CCDS, Rfam, tRNAs & Comparative Genomics)
normalized m6A peaks
66.8888
m6A-Seq
0
Exon 4
(large internal exon)
MSH6
Scale
chr2:
10 kb
hg19
48,015,000
48,020,000
48,025,000
48,030,000
FBXO11
121.857
m6A-Seq
Exon 10
(large internal exon)
BRCA1
Scale
chr17:
41,200,000
41,210,000
41,220,000
41,230,000
41,240,000
41,250,000
41,260,000
41,270,000
NBR2
57.6171
Exon 9
(large internal exon)
ATRX
Scale
chrX:
100 kb
76,800,000
76,850,000
76,900,000
76,950,000
77,000,000
91.3927
Exon 4
(large internal exon)
LATS1
Scale
chr6:
149,985,000
149,990,000
149,995,000
150,000,000
150,005,000
150,010,000
150,015,000
150,020,000
150,025,000
150,030,000
150,035,000
150,040,000
78.8096

Supplementary Figure S2.

(A) Dot blot analysis of m^6A modification levels in poly(A) RNA from the indicated cell lines. (B) Quantification of m^6A dot blot measured by density of dots (n = 2 technical replicates per sample). (C-G) The relative expression of m^6A-modifying enzymes *METTL3* (C), *METTL14* (D), *WTAP* (E), *FTO* (F) and *ALKBH5* (G) in MDA-MB-231 and MCF10A cells, as determined by qPCR (n = 3 technical replicates per cell line). Data in (A, B) are presented as mean ± SD. Data in (C-G) are presented as mean ± SEM.

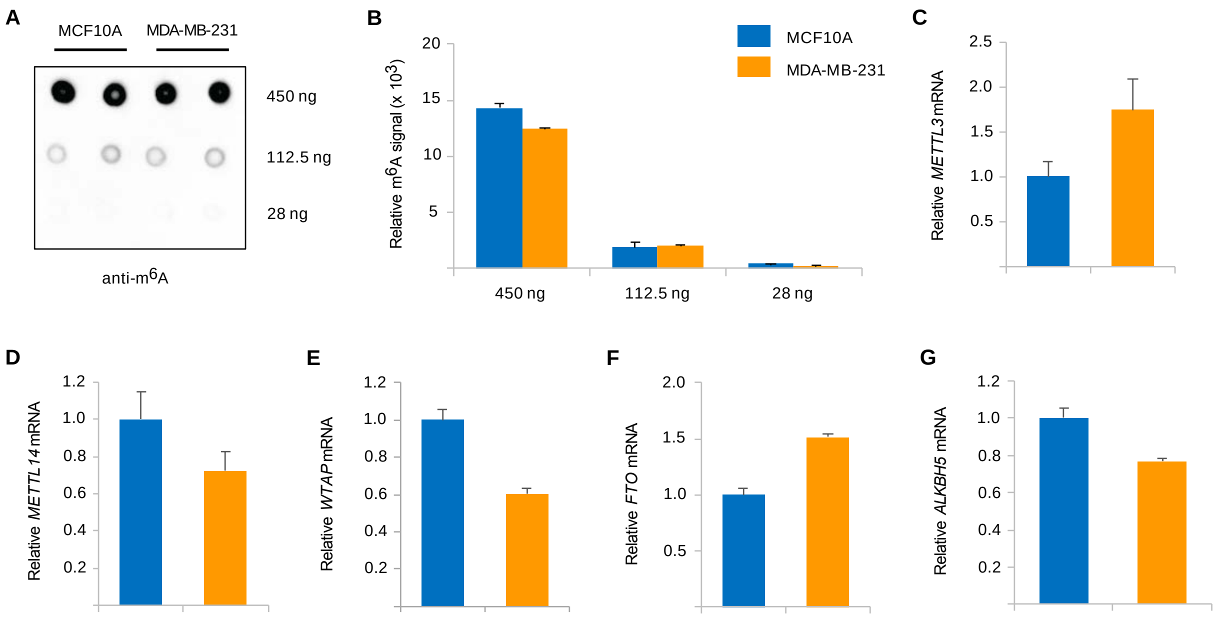
A
MCF10A
MDA-MB-231
450 ng
112.5 ng
28 ng
anti-m6A
B
MCF10A
MDA-MB-231
Relative m6A signal (x 10^3)
20
15
10
5
450 ng
112.5 ng
28 ng
C
Relative METTL3 mRNA
2.5
2.0
1.5
1.0
0.5
D
Relative METTL14 mRNA
1.2
1.0
0.8
0.6
0.4
0.2
E
Relative WTAP mRNA
1.2
1.0
0.8
0.6
0.4
0.2
F
Relative FTO mRNA
2.0
1.5
1.0
0.5
G
Relative ALKBH5 mRNA
1.2
1.0
0.8
0.6
0.4
0.2

Supplementary Table S1. Survey of TSGs for large internal exons and annotated pPA transcripts.

Gene ID	Large Internal Exon (>0.5 kb)	Annotated pPA Isoform	pPA Isoform UCSC ID	Large Internal Exon(s) ID	Large Internal Exon(s) Size (kb)	FL Exons	pPA Exons
APC	no	no				16	
ARID1A	yes	no		18	0.9	20	
ARID1B	yes	no		17	0.8	20	
ARID2	yes	no		15	2.9	21	
ATM	no	no				63	
ATR	yes	no		4	0.9	47	
ATRX	yes	yes	uc010nly.1	9	3.1	35	9
AXIN1	yes	no		9	0.5	11	
BAP1	no	no				17	
BCOR	yes	yes	uc004deq.4	4	2.8	15	4
BRCA1	yes	yes	uc002idd.5	10	3.4	24	10
BRCA2	yes	yes	uc001uua.1	10 | 11	1.1 | 4.9	27	10
CASP8	yes	no		8	0.5	9	
CDKN1A	no	no				4	
CDKN1B	no	no				3	
CDKN2A	no	no				3	
CHEK1	no	no				13	
DAXX	yes	no		3	0.8	8	
DNMT3A	no	no				23	
FAS	no	no				9	
FBXW7	no	no				12	
LATS1	yes	yes	uc003qmw.4	4 | 5	1.5 | 0.6	8	4
LATS2	yes	no		2 | 4 | 5	0.5 | 1.4 | 0.6	8	
MAGI3	yes	yes	n/a	10	0.6	21	10
MAP2K4	no	no				11	
MAP3K1	yes	no		14	1.3	20	
MEN1	no	no				10	
MLH1	no	no				19	
MSH2	no	no				16	
MSH6	yes	yes	uc002rwc.2	4	2.5	10	4
NF1	no	no				58	
NF2	no	no				16	
NOTCH1	yes	no		25	0.6	34	
NOTCH2	no	no				34	
PTEN	no	no				9	
RASA1	no	no				25	
RB1	no	no				27	
RNF43	yes	yes	uc010dcw.3	9	1.4	10	9
SETD2	yes	no		3 | 12 | 15	4.4 | 0.7 | 0.7	21	
SMAD2	no	no				11	
SMAD4	no	no				12	
SMARCA4	no	no				36	
SMARCB1	no	no				9	
STK11	no	no				10	

TET2	no	no			11
TGFBR2	yes	no	5	0.8	8
TP53	no	no			11
TSC1	yes	no	15	0.6	23
TSC2	no	no			42
VHL	no	no			3
WT1	no	no			10
